# Internationales Homöopathisches Jahrhuch

**Published:** 1895-10

**Authors:** 


### BOOK NOTICE.

Internationales Homöopathisches Jahrbuch. Annales homœopathicæ von Dr. Alexander Villers, Volume II. Dresden, 1894. Verlag: Expedition des Homöopathischen Archives, Dr. Alexander Villers. Price, cloth, $1.50 net; by mail, $1.62. For sale by Bœricke & Tafel, 1011 Arch Street, Philadelphia.

This book consists of two parts. The first part contains a list of all the homœopathic physicians in America and Europe, arranged according to countries and cities, and again in alphabetical order, regardless of residence.

The second part is a catalogue of homœopathic literature, arranged alphabetically.

Such a book as this is very useful to every active practitioner as a work of reference. Unfortunately, it has some serious drawbacks. These are errors arising from insufficient proof-reading. There is much bad spelling from this cause, and some addresses are wrongly given. Then there are many omissions. Thus the writer of this review is unable to find his own name and address in the list of physicians in Philadelphia. Others are likewise omitted. All this kind of errors ought to be thoroughly eliminated in future issues, which can be readily done by repeated and careful proof-reading, and by using only the latest and most reliable lists of physicians in making up the volume.

